# Spatial Transcriptomic and Proteomic Analysis Identifies Novel Immune‐Associated Biomarkers of Anti‐PD1 Response in Metastatic Melanoma

**DOI:** 10.1111/sji.70153

**Published:** 2026-10-21

**Authors:** S. A. Koskela, R. E. Kallionpää, I. Mäkelä, E.‐M. Birkman, M. Sundvall, C. R. Figueiredo

**Affiliations:** ^1^ Medical Immuno‐Oncology Research Group, Cancer Research Unit, Institute of Biomedicine, Faculty of Medicine University of Turku Turku Finland; ^2^ Institute of Biomedicine, Faculty of Medicine University of Turku Turku Finland; ^3^ InFLAMES Research Flagship University of Turku Turku Finland; ^4^ Auria Biobank, Turku University Hospital University of Turku Turku Finland; ^5^ Department of Oncology Turku University Hospital Turku Finland; ^6^ Laboratory Division, Department of Pathology, Turku University Hospital University of Turku Turku Finland; ^7^ Cancer Research Unit, Institute of Biomedicine, FICAN West Cancer Centre Laboratories University of Turku Turku Finland; ^8^ Turku Bioscience Center University of Turku Turku Finland

**Keywords:** anti‐PD‐1 resistance, metastatic melanoma, spatial transcriptomics

## Abstract

Resistance to anti‐PD1 therapy remains a major challenge in metastatic melanoma, despite long‐term benefit in some patients. Since a functional dendritic cell (DC)‐CD8^+^ T cell axis is essential for anti‐PD1 response, biomarkers associated with this axis may help characterize underexplored immune states linked to resistance. Here, we further characterized poor survival predictor (PSP) biomarkers previously negatively correlated with DC‐CD8^+^ T cell axis in melanoma, evaluating their associations with anti‐PD1 response, immune infiltration, and transcriptional and proteomic signatures. Pre‐treatment transcriptomic data were analysed using CIBERSORTx and Reactome, while NanoString GeoMx spatial transcriptomic and proteomic data were used to resolve biomarker‐associated features across tumour and immune compartments. We identified 28 PSP biomarkers significantly increased in non‐responsive patients with strong correlations with one another, suggesting coordinated transcriptional patterns within a tumour microenvironment unfavourable to therapeutic response and survival. These biomarkers were associated with immunosuppressive cell types, decreased cytotoxicity, and pathways related to transcription, translation, epigenetic regulation, and signal transduction. Spatially, PSP biomarkers were expressed in both tumour and immune compartments, but survival associations were compartment‐ and site‐specific. Among them, *FAM57A*, *GSTA4* and *KCTD1* were associated with poor survival when highly expressed in the CD45^+^ immune compartment of lymph node metastases. Their expression was associated with transcriptomic signatures of reduced immune activation, altered antigen presentation, and epigenetic regulation, and increased proteomic PI3K‐AKT‐mTORC1/MAPK signalling, especially upon co‐expression. Together, these results identify *FAM57A*, *GSTA4* and *KCTD1* as emerging immune‐associated biomarkers linked to established resistance‐associated immune states and poor prognosis in metastatic melanoma.

## Graphical abstract

Integrated anti‐PD1 transcriptomic datasets identified poor survival predictor biomarkers associated with therapy resistance in metastatic melanoma. Spatial transcriptomic and proteomic profiling revealed *FAM57A*, *GSTA4*, and *KCTD1* as immune‐associated biomarkers linked to altered immune transcriptomic signatures, increased PI3K‐AKT‐mTORC1/MAPK proteomic signalling, and poor clinical outcome.

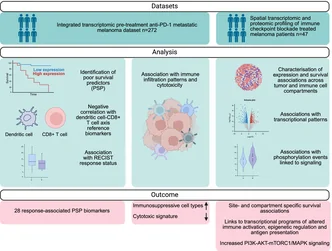

**Abbreviations**
anti‐PD1: anti‐programmed cell death protein 1
CR: complete response
CTLA‐4: cytotoxic T‐lymphocyte‐associated protein 4
DC: dendritic cell
EGA: European Genome‐phenome Archive
ENA: European Nucleotide Archive
FFPE: formalin‐fixed paraffin‐embedded
GEO: Gene Expression Omnibus
GSEA: Gene Set Enrichment Analysis
ICT: immune checkpoint therapy
irRECIST: immune‐related Response Evaluation Criteria in Solid Tumours
MAPK: mitogen‐activated protein kinase
MR: mixed response
MSigDB: Molecular Signatures Database
mTORC1: mechanistic target of rapamycin complex 1
NE: not evaluable
NGS: next‐generation sequencing
OS: overall survival
PD: progressive disease
PI3K: phosphoinositide 3‐kinase
PR: partial response
PSP: poor survival predictor
RECIST: Response Evaluation Criteria in Solid Tumours
ROI: region of interest
SRA: Sequence Read Archive
TCGA‐SKCM: the cancer genome atlas skin cutaneous melanoma
TMA: tissue microarray
TMB: tumour mutational burden
TME: tumour microenvironment
TPM: transcripts per million

## 1 Introduction

Immune checkpoint therapies (ICT) have transformed cancer treatment by targeting inhibitory receptors such as CTLA‐4 and PD‐1, thereby restoring the cytotoxic activity of CD8^+^ T cells against tumour cells and leading to remarkable clinical efficacy in several advanced solid cancers [1]. Although approximately half of the metastatic melanoma patients treated with anti‐PD1 treatment reach 10‐year overall survival, half of the patients do not benefit from anti‐PD1 due to primary and acquired resistance mechanisms [2, 3]. These resistance mechanisms pose a significant challenge, increasing clinical and societal side effects of ineffective treatments.

In metastatic melanoma, resistance to anti‐PD1 therapy has been linked to several factors, including low tumour mutational burden (TMB) and antigen load, impaired antigen presentation, epigenetic alterations, T cell dysfunction or exclusion, deficient IFN‐γ signalling, as well as clinical variables such as age and tumour stage [4, 5, 6, 7, 8, 9, 10]. However, these factors capture only individual aspects of anti‐tumour immunity, whereas the complexity of the tumour–immune microenvironment (TME) calls for integrative approaches that consider multiple immune effector functions simultaneously [11]. Because CD8^+^ T cells are the primary effectors reinvigorated by anti‐PD1 therapy [12, 13, 14], understanding the pathways that regulate their activation and function is particularly important. Effective CD8^+^ T cell–mediated anti‐tumour immunity is initiated in secondary lymphoid organs, where dendritic cells (DCs) present tumour antigens to naïve T cells, providing antigen presentation, co‐stimulatory signals, and cytokines required for T cell priming [15]. Following subsequent T cell clonal expansion and migration into the TME, DCs support the differentiation and cytotoxic functions of CD8^+^ T cells in TME and are critical determinants of responses to ICT [16, 17, 18, 19]. Therefore, a functional DC‐CD8^+^ T cell axis is essential for anti‐PD1 response.

Although recent studies have linked DC functionality to anti‐PD1 responses in metastatic melanoma patients [20, 21], biomarker‐associated features of the DC‐CD8^+^ T cell axis remain incompletely characterized. Using a systems‐level transcriptomic approach, we previously identified 668 good and 129 poor survival predictors that correlate positively or negatively, respectively, with *CD74* and *CD8A* representing the DC‐CD8^+^ T cell axis [22, 23]. These biomarkers were further refined into a 15‐gene signature predictive of response in patients with high CD8^+^ T cell infiltration, consistent with transcriptional programs associated with effective antitumor immune responses. Notably, the roles of many poor survival predictors (PSP) in antitumor immunity remained unknown or unclear [22, 23].

The current study extends our previously reported findings by spatially characterizing response‐associated PSP biomarkers in tumour and immune cell compartments of metastatic melanoma. Among the 28 PSP biomarkers linked to non‐response, *FAM57A*, *GSTA4* and *KCTD1* were specifically associated with clinical outcomes when expressed in the immune cell compartment of melanoma lymph node metastases. Spatial profiling of the whole transcriptome and multiple NanoString GeoMX proteome panels revealed that these biomarkers are associated with transcriptional and proteomic programs consistent with reduced immune activation, altered antigen‐presentation‐related features, and increased PI3K‐AKT‐mTORC1/MAPK signalling. Thus, the novelty of this study lies in linking poorly characterized PSP biomarkers to immune compartments characterized by established molecular features of anti‐PD1 resistance. These compartment‐specific associations highlight the importance of spatial context in defining the clinical relevance of PSP biomarkers and underscore the need to resolve their cell‐type–specific expression patterns.

## 2 Materials and Methods

### 2.1 Patients and Samples

Four metastatic melanoma cohorts treated with anti‐PD‐1 monotherapy or anti‐PD‐1–based combination therapy [8, 24, 25, 26] with publicly available transcriptomic and clinical data were integrated into a combined dataset as described previously [27]. RNA‐seq datasets from pre‐treatment samples, days of survival, death events and response criteria from patients receiving anti‐PD1 were collected from anti‐PD1 Liu cohort [24], anti‐PD1 Hugo cohort [8], anti‐PD1 Riaz cohort [26], anti‐PD1/anti‐CTLA4 Gide cohort [25] available at Gene Expression Omnibus (GEO), Sequence Read Archive (SRA), European Nucleotide Archive (ENA), European Genome‐phenome Archive (EGA) and database of Genotypes and Phenotypes (dbGaP). Clinical responders (Complete Response; CR, Partial Response; PR) were distinguished from non‐responders (Stable Disease; SD, Progressive Disease; PD) using irRECIST or RECIST v1.1 criteria as reported in the original publications. Patients with not evaluable (NE) or mixed (MR) response status were excluded from response analyses. irRECIST criteria were applied in Hugo [8] cohort, while RECIST v1.1 criteria were used to stratify patients in Liu [24], Gide [25] and Riaz [26].

In addition, Tissue MicroArray (TMA) comprising 141 primary and metastatic melanoma samples from a total of 47 patients was built in collaboration with Auria Biobank (Turku, Finland). Of those, lymph node metastasis samples and skin metastasis samples from 38 patients were used to evaluate survival effect and molecular associations of the Poor Survival Predictors in tumour and immune compartments using NanoString GeoMX proteome and whole transcriptome analyses. The use of tissue samples was approved by the Scientific Steering Group of Auria Biobank (decision AB22‐4803, granted on 25.4.2022). The study and clinical data collection was approved by the Institutional Review Board of Turku University Hospital (2025‐0692‐TL; T695/2025). Briefly, TMA was constructed from archived formalin‐fixed paraffin‐embedded (FFPE) melanoma samples from patients who had a histologically confirmed diagnosis of metastasized cutaneous melanoma in the Turku University Hospital between the years 2005 and 2022. Patients and biopsy regions were selected using electronic health records and H&E pathological evaluation to focus on areas where immune and tumour compartments intersect.

### 2.2 Bulk RNA Sequencing Data Processing

The downloaded SRA files were converted into FASTQ files using sratoolkit (2.11.0). The quality of the sequenced reads was observed using FastQC (v0.11.9) tool (https://www.bioinformatics.babraham.ac.uk/projects/fastqc/). Transcript per Million (TPM) values were quantified using the human reference genome (GRCh38) and Kallisto (v0.48.0) (https://pachterlab.github.io/kallisto/). The TPM values of all anti‐PD1 cohorts were adjusted for batch correction using the R package ComBat‐seq [28] to create the integrated anti‐PD1 dataset (*n* = 272). The TPM values for the Liu dataset are available in Supporting Information of the original publication [24].

### 2.3 Defining Poor Survival Predictors

The pipeline for identification of Poor Survival Predictors has been published previously [22, 23]. Briefly, the association between whole transcriptome gene expression and patient survival was assessed in integrated metastatic melanoma anti‐PD1 dataset and Genomic Data Commons—The Cancer Genome Atlas—Skin Cutaneous Melanoma (GDC–TCGA–SKCM) melanoma dataset (downloaded from Xena Functional Genomic Explorer of the University of California, Santa Cruz https://xenabrowser.net/heatmap/) using log‐rank test. The expression of genes with a statistically significant association with poor survival was correlated with *CD74* and *CD8A* to identify biomarkers that correlate with the DC‐CD8^+^ T cell axis. Significantly negatively correlated genes were defined as poor survival predictors.

### 2.4 Spatial NGS Transcriptomic Analysis From Metastatic Melanoma Tumours

Gene expression patterns of PSP biomarkers and the whole transcriptome were assessed using the metastatic melanoma TMA from Auria Biobank. When preparing the TMA, a representative site of the paraffin block of each tumour was punched with a 1 mm diameter cylinder, and an automated tissue arrayer (TMA Grand Master, 3DHISTECH Ltd., Budapest, Hungary) was used. The samples were provided for NanoString spatial Next Generation Sequencing (NGS) analysis as 3.5 μm TMA sections focusing on immune infiltrated areas. Whole transcriptome analysis of cutaneous melanoma biopsies from metastatic melanoma patients was conducted using the NanoString Spatial GeoMX Whole Transcriptome Atlas RNA probe kit (NanoString Technologies, Seattle, WA, USA) following the manufacturer's guidelines with some modifications. In brief, the following workflow was used: deparaffinization of 3.5 μm‐thick FFPE sections, antigen retrieval, and overnight RNA probe hybridization.

### 2.5 Spatial NGS Proteomic Analysis From Metastatic Melanoma Tumours

Associations of *FAM57A*, *GSTA4* and *KCTD1* with the expression of multiple immune function and signalling proteins were assessed in the CD45^+^ immune compartments of metastatic melanoma TMA. NanoString GeoMX Spatial Distribution Analysis was performed for the protein targets following the manufacturer's guidelines. In brief, the following workflow was used: deparaffinization of 3.5 μm‐thick FFPE sections, antigen retrieval, and overnight antibody incubation of detection (Immune Cell Profiling Panel, Myeloid Panel, Immune Activation Status Panel, IO Drug Target Panel, MAPK Signalling Panel, PI3K/AKT Signalling Panel, NanoString Technologies) and morphology markers (Human Melanoma TME morphology kit, NanoString Technologies).

### 2.6 Region of Interest (ROI) Selection and Analysis

Both transcriptomic and proteomic analysis slides were stained using the Human Melanoma TME morphology kit's antibodies (NanoString Technologies) to identify Regions of Interest (ROIs). The kit enables the identification of areas predominantly occupied by tumour cells (PMEL17^+^/S100B^+^ green) and immune cells (CD45^+^ red). SYTO13 stain was used to visualize nuclei. The staining was visualized on the NanoString GeoMX instrument. Morphology markers PMEL17^+^/S100B^+^ (green) and CD45^+^ (red) were used for detection and selection of tumour and immune regions of interest (ROIs), respectively. Immune cells near the interface with tumour cells were prioritized. RNA‐specific probes (transcriptome analysis) and photocleavable oligonucleotide tags attached to detection antibodies (proteome analysis) were cleaved from tumour and immune ROIs and prepared for next‐generation sequencing. All data were processed and normalized using NanoString GeoMX DSP Analysis Suite and manufacturer's recommendations. The transcriptome data were filtered to exclude ROIs with less than 5% of genes detected above the limit of quantification and to exclude genes not detected above the limit of quantification in at least 5% of the segments. Data were normalized according to the Quartile 3 method. The proteome data were normalized to the geometric mean of housekeeping proteins Histone H3, GAPDH and S6 expression levels.

### 2.7 Quantification of Immune Infiltration

Abundance of tumour‐infiltrating immune cell subsets in the integrated anti‐PD1 bulk‐RNAseq dataset was estimated by imputing cell fractions using the LM22 signature matrix in the CIBERSORTx deconvolution algorithm with B‐mode batch correction and 1000 permutations [29]. Immune cell abundances in the CD45^+^ immune compartments of the local anti‐PD1 dataset were estimated using the SpatialDecon algorithm (NanoString) [30].

### 2.8 Network and Pathway Analyses

Associations of 28 response‐significant PSP biomarkers with different pathways were analysed using Reactome V92 (https://reactome.org). The number of PSP biomarker hits was summed for each pathway, and the top 20 pathways with the highest counts were plotted using the circlize package in R. Full protein–protein interaction networks including physical and functional protein associations of FAM57A, GSTA4 and KCTD1 were determined by an online tool, STRING v.12.0 (https://string‐db.org). The minimum required interaction score was set to high confidence (0.700).

### 2.9 Transcriptomic Analyses

Differential gene expression analysis was performed on the CD45^+^ immune compartment in lymph node metastatic melanoma biopsies from local anti‐PD1 dataset. Patients were stratified into high‐ and low‐expression groups for selected biomarker genes (*FAM57A*, *GSTA4* and *KCTD1*) using gene‐specific expression cutoffs determined from survival‐based thresholds. For each comparison, log_2_ fold changes were calculated as the ratio of mean expression between high‐ and low‐expression groups. Statistical significance was assessed using two‐sided *t*‐tests on gene‐wise expression values across groups. Genes were considered differentially expressed if they met both a nominal *p*‐value threshold of < 0.05 and an absolute log_2_ fold change > 0.5. Volcano plots were generated to visualize the distribution of differentially expressed genes, with the most significantly upregulated and downregulated genes annotated. Up and downregulated genes were analysed using Reactome V92 (https://reactome.org). Furthermore, Gene Set Enrichment Analysis was performed on high and low PSP biomarker expression samples using Hallmarks gene set (h.all.v2025.1.Hs.symbols.gmt) from Molecular Signatures Database (MSigDB, https://www.gsea‐msigdb.org/gsea/index.jsp).

### 2.10 Statistical Analysis

GraphPad Prism 8.3.1 (GraphPad) software was used for statistical analysis. Statistical tests were guided by normal distribution tests using the Shapiro–Wilk normality test performed for all data sets. Data sets with non‐normal distribution were analysed with unpaired non‐parametric two‐tailed Mann–Whitney *U* test and non‐parametric two‐tailed Spearman's correlation test. Differences were considered significant when *p* < 0.05 (*), *p* < 0.01 (**), *p* < 0.001 (***) and *p* < 0.0001 (****). Overall survival was evaluated with Kaplan–Meier plots and Log‐rank test by evaluating thresholds within the 0.25–0.75 quartile range and selecting the one that yielded the lowest log‐rank *p*‐value, ensuring the best separation between survival curves. The high and low group annotations defined in the survival analysis for *GSTA4*, *FAM57A* and *KCTD1* were applied throughout the article. Statistical significance for analyses involving multiple targets was assessed using multiple *t*‐tests with FDR correction.

## 3 Results

### 3.1 Identification of Poor Survival Predictors Associated With Anti‐PD1 Resistance and Coordinated Regulatory Programs

To elucidate the roles of the PSP biomarkers, identified previously [22, 23], in antitumor immune response and anti‐PD1 immunotherapy, we assessed previously integrated [27] transcriptomic and clinical data from pre‐treatment biopsies across multiple cohorts (Liu, Gide, Riaz, Hugo), all including at least 5‐year overall survival and response evaluated by irRECIST or RECIST v1.1 criteria [8, 24, 25, 26]. Consistent with the DC‐CD8^+^ T cell axis framework, high expression of these biomarkers correlated with poor survival outcomes and inversely with *CD74* and *CD8A* expression [22, 23] (Figure 1A), linking them to the DC‐CD8^+^ T cell axis. To filter the biomarkers related to the anti‐PD1 objective response, their expression was evaluated between anti‐PD1 responders (Complete Response, Partial Response) and non‐responders (Stable Disease, Progressive Disease). The expression of 28 PSP biomarkers, namely *ADCY6*, *ANKS6*, *ARHGAP42*, *BEND3*, *CCDC140*, *CHST10*, *FAM171A2*, *FAM189B*, *FAM210B*, *FAM57A*, *FBXO17*, *GPSM1*, *GSTA4*, *HOXA11*, *HPDL*, *IFT22*, *KCTD1*, *KCTD15*, *MEIS3P2*, *PROSER2*, *RCOR2*, *TIMM13*, *TUB*, *URB1*, *USP22*, *ZNF470*, *ZNF584* and *ZNF623* was significantly increased in non‐responders (Figure 1A, Figure S1), aligning with the survival association. The expression of these biomarkers also showed strong positive correlations with one another (Figure 1B), suggesting that, even if they are not involved in the same pathways, they may participate in distinct pathway‐associated programs that coexist within a TME unfavourable to therapeutic response and survival. Reactome pathway mapping of the 28 PSP biomarkers revealed that the biomarkers were predominantly linked to transcription, translation, epigenetic regulation, and signal transduction‐related pathways as the most represented pathway categories (Figure 1C). Together, these results identify 28 PSP biomarkers associated with unfavourable response to anti‐PD1 and enriched in pathway categories related to epigenetic, transcriptional, and translational regulation.

**FIGURE 1:**
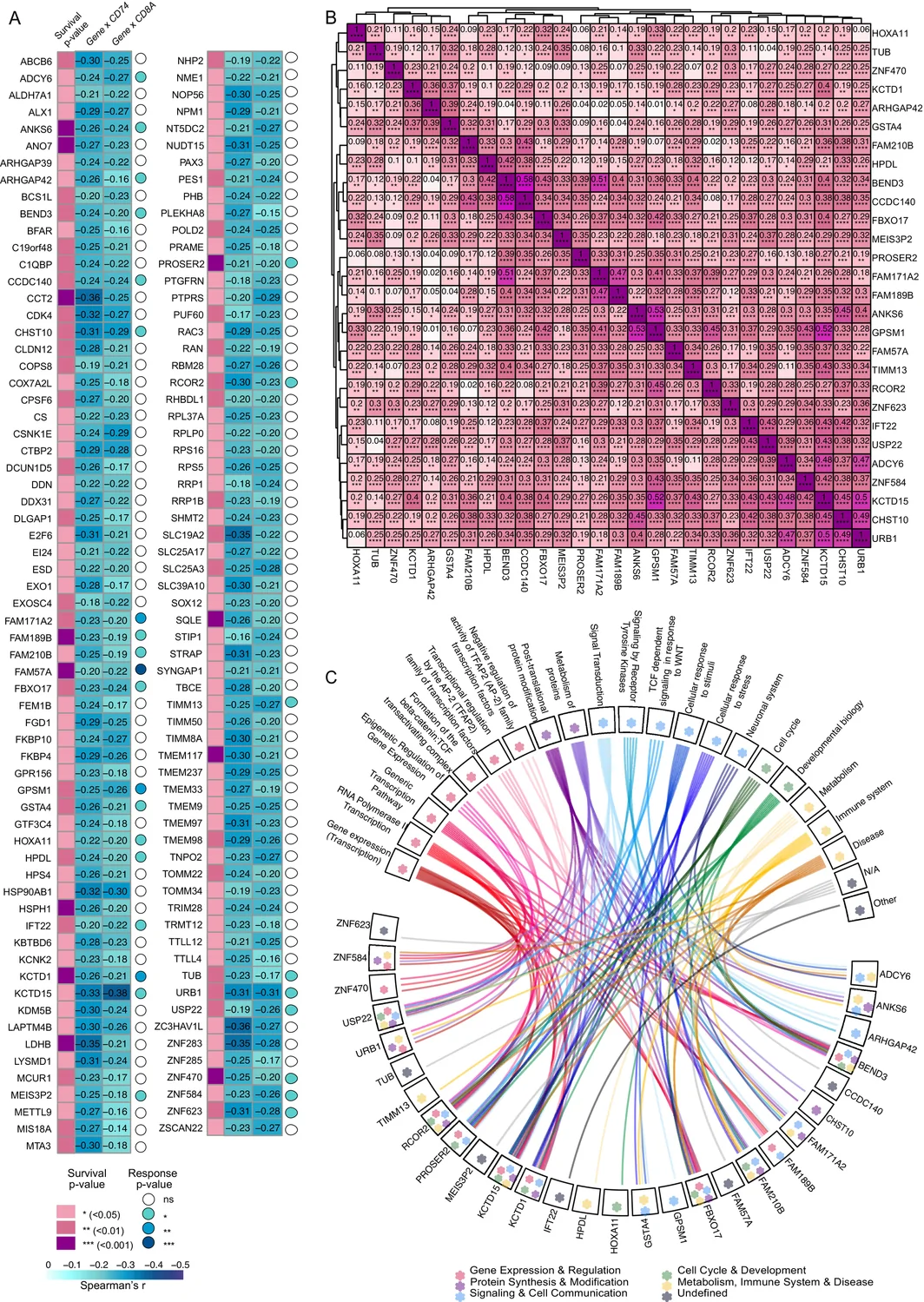
Poor survival predictor biomarkers (*n* = 28) linked to transcriptional regulation, signalling and metabolic pathways are associated with anti‐PD1 objective response. (A) Heatmaps showing the association of poor survival predictor biomarkers (PSP [22, 23]) with patient survival, and their correlation with *CD74* and *CD8A*, reference biomarkers for DC‐CD8^+^ T cell axis, in anti‐PD1 pre‐treatment samples from four integrated publicly available metastatic melanoma cohorts (*n* = 272). The association of each PSP biomarker with objective response, defined by RECIST v1.1 or irRECIST criteria, was analysed with either unpaired *t*‐test or Mann–Whitney *U*‐test, depending on the dataset distribution, evaluated by Shapiro–Wilk test. (B) Heatmap showing Spearman's correlation between the gene expression of PSP biomarkers in integrated anti‐PD1 dataset. (C) The Reactome pathway associations of the 28 PSP biomarkers were analysed using Reactome platform (https://reactome.org). The total number of matched entities and interactors was used to highlight the pathways with which most biomarkers are associated. Top 20 pathways and their associations with each biomarker are highlighted using the CIRCOS plot.

### 3.2 Poor Survival Predictors Are Associated With Suppressive Immune Infiltration and Decreased Cytotoxicity

The 28 PSP biomarkers inversely correlated with the DC‐CD8^+^ T cell axis reference biomarkers *CD74* and *CD8A* but their roles in immunity remain poorly characterized. Although *CD74* is expressed by multiple antigen‐presenting cell types and may predominantly reflect B cells in lymph nodes, it was used here as a correlative reference marker for the DC‐CD8^+^ T‐cell axis due to its predominant correlation with DC infiltration in the tumour rather than as a DC‐specific marker. To explore their immune associations, we used the CIBERSORTx deconvolution algorithm on the integrated anti‐PD1 transcriptomic dataset to estimate immune cell fractions in the TME and correlate PSP biomarker gene expression with immune cell abundances. Spearman correlation coefficients between PSP biomarker expression and immune cell fractions were clustered to reveal patterns (Figure 2A), identifying six distinct immune cell‐type clusters. Clusters were subsequently annotated based on the dominant functional phenotypes of the constituent immune cell types. Clusters 1 and 2 (C1, C2), comprising immunosuppressive myeloid regulators and proinflammatory mediators, showed clear positive and negative correlations, respectively. This suggests that most biomarkers are positively associated with the abundance of immunosuppressive cell types, such as M2 macrophages, as well as monocyte populations that may contribute to immunosuppressive phenotypes in the TME, and negatively with the abundance of proinflammatory populations like CD8^+^ T cells, activated CD4^+^ memory T cells, M1 macrophages, and B cells. Correlation patterns were less distinct for cell types in the helper‐regulator axis, innate immune responders, resting effectors, and innate‐adaptive interface clusters (Figure 2A). Among the PSP biomarkers originally identified through a DC‐CD8^+^ T cell axis‐focused pipeline, *ARHGAP42*, *ZNF584*, *ADCY6*, *URB1*, *CHST10* and *IFT22* negatively correlated with CD8^+^ T cell frequencies. *IFT22*, *FAM57A*, *FAM171A*, and *FAM189B* showed negative correlations with resting dendritic cell frequencies, and none correlated with the frequency of activated dendritic cells (Figure 2A). However, all biomarkers except *HOXA11* correlated with cytotoxic molecule expression and the cytolytic score (CTL score), defined as the average expression of *CD8A*, *CD8B*, *GZMA*, *GZMB* and *PRF1* [9] (Figure 2B), suggesting that, despite limited association with the abundance of CD8^+^ T cell infiltration, their expression is associated with an immunosuppressive TME with reduced cytotoxic activity.

**FIGURE 2:**
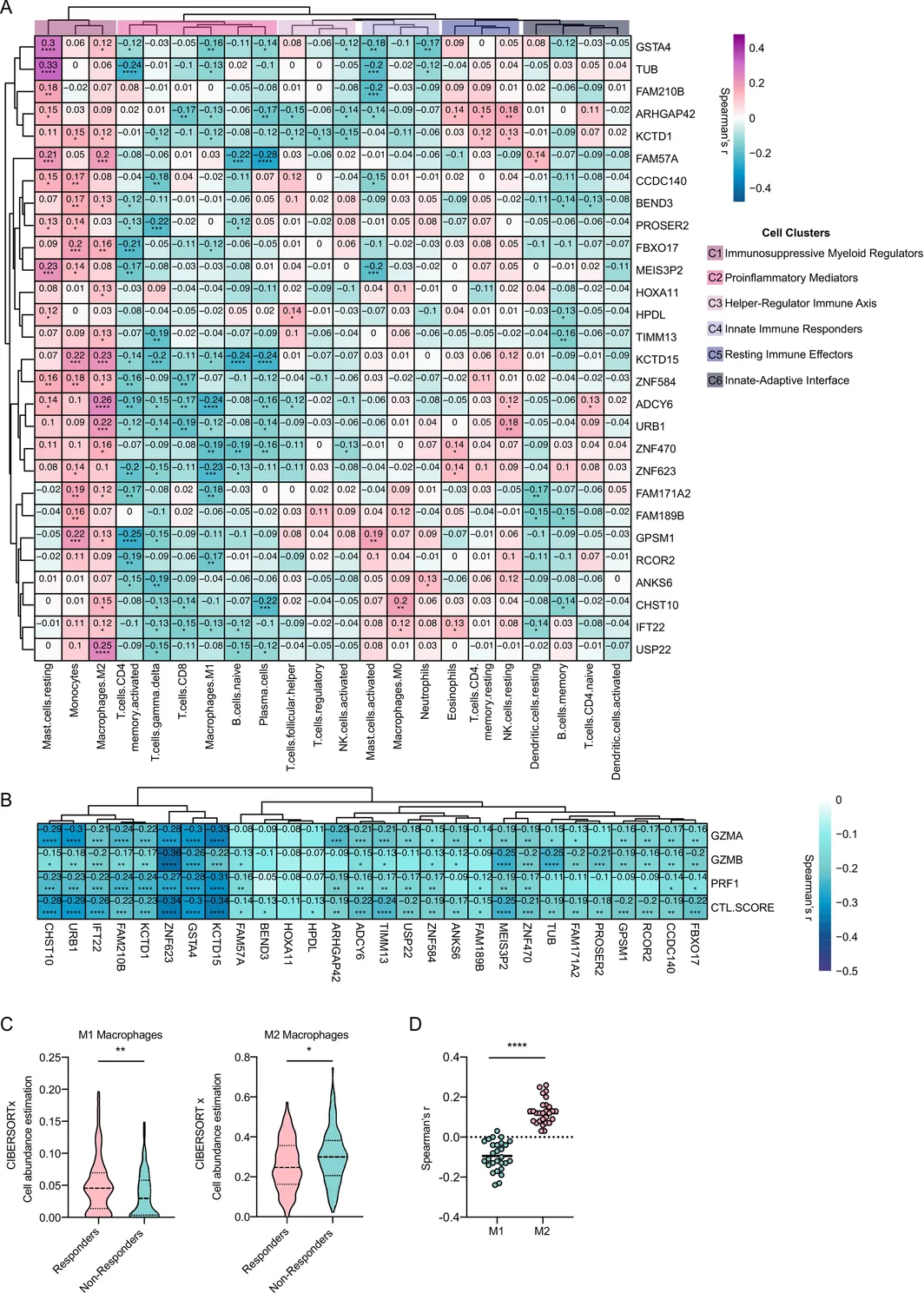
Poor survival predictors are associated with suppressive immune infiltration and decreased cytotoxicity. (A) Heatmaps showing Spearman's correlation of PSP mRNA expression and immune cell abundance estimated by CIBERSORTx in integrated anti‐PD1 dataset (*n* = 272). (B) Heatmap showing Spearman's correlation of the PSP gene expression with cytotoxic components of CTL score [9] as well as the CTL score (average expression of *CD8A*, *CD8B*, *GZMA*, *GZMB* and *PRF1*) in integrated anti‐PD1 dataset (*n* = 272). (C) Immune cell abundance estimate levels estimated by CIBERSORTx in responders (Complete Response, Partial Response, *n* = 112) and non‐responders (Stable Disease, Progressive Disease, *n* = 156). (D) Spearman's correlation of PSP gene expression with estimated infiltration of M1 and M2 macrophages. Each dot represents the correlation of one PSP gene with the estimated infiltration.

To better understand how these biomarkers associate with response through immune mechanisms, we first evaluated which immune infiltrates were associated with response. Notably, only M1 and M2 macrophage frequencies differed significantly between responders and non‐responders (Figure 2C, Figure S2A), despite known roles of other cell types in anti‐PD1 outcomes [31]. Moreover, biomarker correlations with M1 and M2 abundances differed significantly (Figure 2D), suggesting an association with M1/M2 balance and immunogenicity‐related features in the TME.

### 3.3 The Biomarkers Are Equally Expressed in Tumour and Immune Cell Compartments but Display Differential Survival Patterns

To assess whether the associations of these biomarkers within the TME are linked to immune cell‐ or tumour cell‐specific compartments, we performed NanoString GeoMX spatial transcriptomic profiling on a local metastatic melanoma dataset. The cohort included metastatic melanoma samples from patients treated in a real‐world clinical setting, including immune checkpoint blockade and other treatments administered at different lines of therapy (Table 1). A tissue microarray (TMA) consisting of melanoma biopsies from 42 lymph node metastases (31 patients) and 16 skin metastases (14 patients) was stained for S100B/PMEL17 to identify tumour cell compartment and for CD45 to identify immune cell compartment. We first assessed overall survival associations for the 28 biomarkers in the tumour and immune cell compartments of skin/subcutaneous and lymph node metastases. Notably, only the lymph node immune compartment showed statistically significant survival associations for a subset of the biomarkers, while no significant associations were observed in the skin metastases immune compartments (Figure 3A). Based on this, we focused subsequent analyses on lymph node biopsies.

**TABLE 1:** Patient characteristics.

	Mean (range)
Age (years) at initial diagnosis of melanoma	63 (28–81)
	Number of patients (%)
Sex	
Female	14 (37%)
Male	24 (63%)
BRAF‐V600 mutation status	
WT	18 (47%)
Mutated	12 (32%)
Unknown	8 (21%)
Primary diagnosis: Stage	
I	2 (5%)
IIA	1 (3%)
IIB	4 (11%)
IIIA	3 (8%)
IIIB	6 (16%)
IIIC	13 (34%)
IV	6 (16%)
Unknown	3 (8%)
Metastasis sites at the diagnosis of metastatic disease	
Lymph node	23 (60%)
Visceral	14 (37%)
Brain	1 (3%)
Skin/subcutaneous	16 (42%)
Bone	5 (13%)
Other	4 (11%)
Adjuvant treatment	
No	26 (68%)
Ipilimumab	1 (3%)
Nivolumab	5 (13%)
Interferon	1 (3%)
Dabrafenib + Trametinib	2 (5%)
Radiotherapy	1 (3%)
Metastatic disease: treatment at any line	
Ipilimumab	2 (5%)
Ipilimumab + Nivolumab	4 (11%)
Nivolumab	17 (44%)
Pembrolizumab	8 (21%)
Encorafenib + Binimetinib	5 (13%)
Dabrafenib + Trametinib	6 (16%)
Karboplatin + Paclitaxel	10 (26%)
Karboplatin + Etoposide	1 (3%)
Dacarbazine‐based chemotherapy regimen	4 (11%)
Temodal or Temodal–Vincristin–Lomustin	9 (24%)
Operative care	14 (37%)
Radiotherapy	19 (50%)
T‐Vec (Talimogene Laherparepvec)	2 (5%)
Isolated limb perfusion	3 (8%)
MATINS trial (Bexmarilimab)	1 (3%)
Nivolumab + Relatlimab	2 (5%)

**FIGURE 3:**
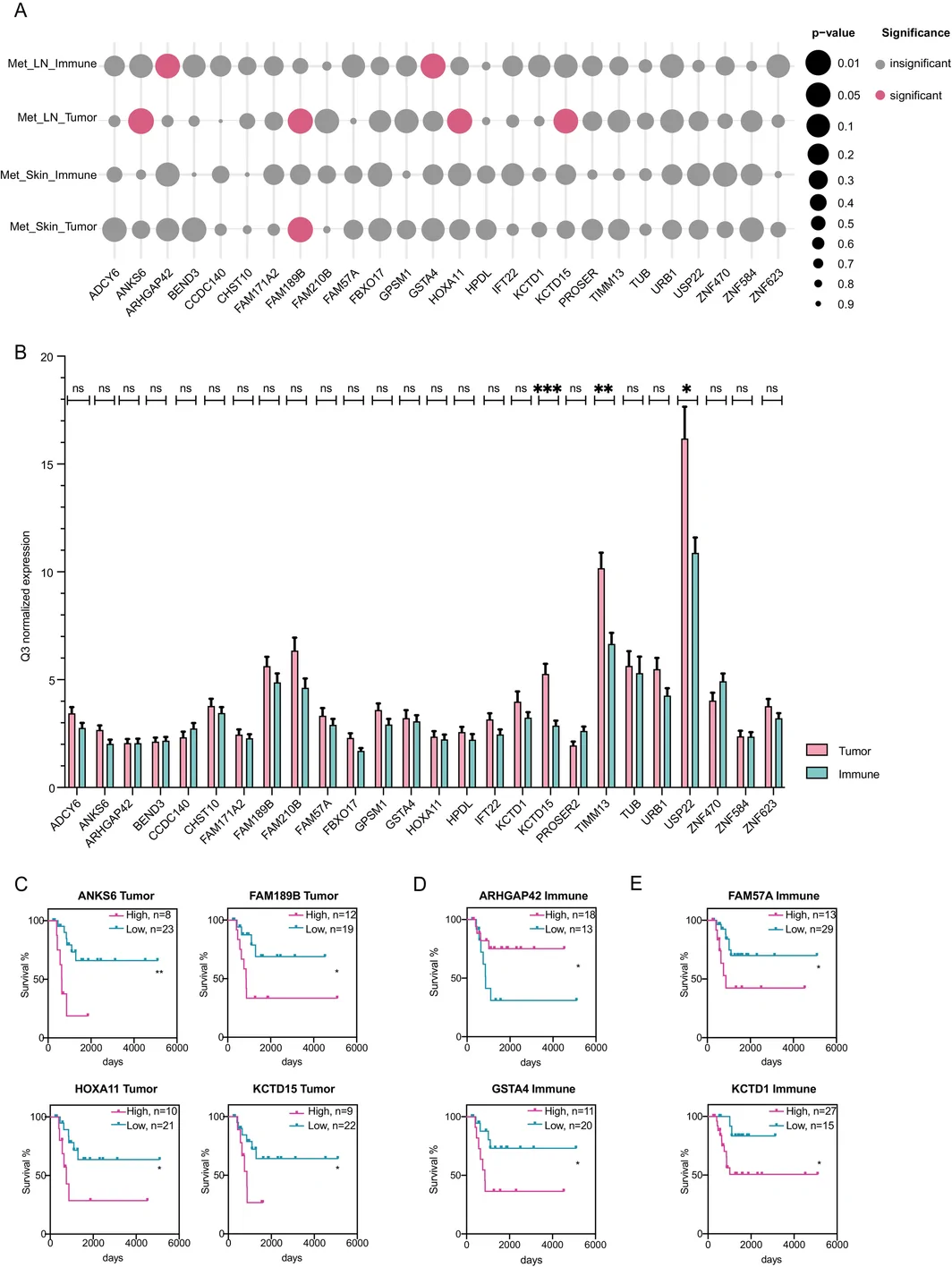
The biomarkers are expressed in both tumour and immune compartments but display differential survival patterns. (A) Association of PSP biomarker mRNA expression with patient survival in tumour cell (S100B/PMEL17^+^) and immune cell (CD45^+^) compartments of metastatic melanoma lymph node biopsies analysed using NanoString GeoMX and log‐rank test. (B) PSP gene expression patterns in tumour (S100B/PMEL17^+^) and immune (CD45^+^) compartments of metastatic melanoma lymph node biopsies analysed using NanoString GeoMX. The difference in expression patterns between compartments were analysed with either unpaired *t*‐test or Mann–Whitney *U*‐test, depending on the dataset distribution, evaluated by Shapiro–Wilk test. (C) Kaplan–Meier survival plots of PSP biomarkers that are statistically significantly associated with survival when expressed in the tumour compartment of 31 lymph node metastatic melanoma biopsies from 31 patients. (D) Kaplan–Meier survival plots of PSP biomarkers that are statistically significantly associated with patient survival when expressed in the immune compartment of 31 metastatic melanoma lymph node biopsies from 31 patients. (E) Kaplan–Meier survival plots of PSP biomarkers that are statistically significantly associated with patient survival when expressed in the immune compartment of 42 lymph node metastatic melanoma biopsies from 31 patients.

Next, the expression of the 28 selected PSP biomarkers was compared between lymph node tumour and immune cell compartments. Most biomarkers were expressed in both compartments, with a general trend toward higher expression in the tumour cell compartment (Figure 3B), indicating that the majority of the biomarkers are not exclusively immune‐ or tumour‐intrinsic but may act across both compartments. *KCTD15*, *TIMM13* and *USP22* were statistically significantly enriched in the tumour cell compartment (Figure 3B).

Survival analysis within each cell compartment revealed distinct compartment‐specific associations between biomarker expression and survival outcomes. High expression levels of *ANKS6*, *FAM189B*, *HOXA11* and *KCTD15* in the tumour compartment were also significantly associated with poorer survival in this new dataset (Figure 3C). When considering the earliest available biopsy from diagnosis for each patient, the high expression level of *ARHGAP42* in the immune compartment was surprisingly associated with improved survival, while the expression of *GSTA4* was associated with poorer survival (Figure 3D). Although the survival pattern of *ARHGAP42* contradicted the original hypothesis of poor survival biomarkers, it displays the opposite behaviour in the tumour cell compartment, as it was negatively but insignificantly associated with survival in the tumour compartment (Figure S3A). This suggests that the poor survival association of *ARHGAP42* in the bulk‐RNA dataset may have originated from higher expression in tumour cells, potentially contributing to the observed discrepancy.

When considering all available lymph node metastatic melanoma biopsies, expression of *FAM57A* and *KCTD1* was also statistically significantly associated with survival in the immune compartment (Figure 3E). This trend persisted when considering the earliest available biopsy from diagnosis (Figure S3B). Although the association of high *FAM57A* and *KCTD1* expression with poor survival remains borderline significant in the immune cell compartment of this dataset, their roles in immunity and melanoma anti‐PD1 responses remain largely unexplored. Together with *GSTA4*, which displayed a statistically significant association with survival, these biomarkers were prioritized for further characterization because of their immune cell compartment‐specific survival association and relatively limited characterization in the context of tumour immunity. We therefore sought to further characterize their immune‐related associations.

### 3.4 *FAM57A* , *GSTA4* And *KCTD1* Expression in Immune Compartment Is Not Associated With Altered Immune Infiltration Patterns

As CIBERSORTx analysis of the bulk RNA‐seq data linked the biomarkers to immunosuppressive cell types, we next investigated immune cell abundancies and their association with biomarker expression within the immune compartment. Interestingly, the abundance of immune cell types, estimated by SpatialDecon algorithm, did not correlate with or relate to the expression level or status of any biomarker (Figure 4A,B, Table S1). For the exploratory comparisons, the samples were treated as separate observations, and multiple samples from the same patient were considered separately. There was a trend of increased naïve B cell, memory B cell, memory CD4^+^ and CD8^+^ T cells and natural killer (NK) cell levels in the biomarker low expression groups (Figure 4B).

**FIGURE 4:**
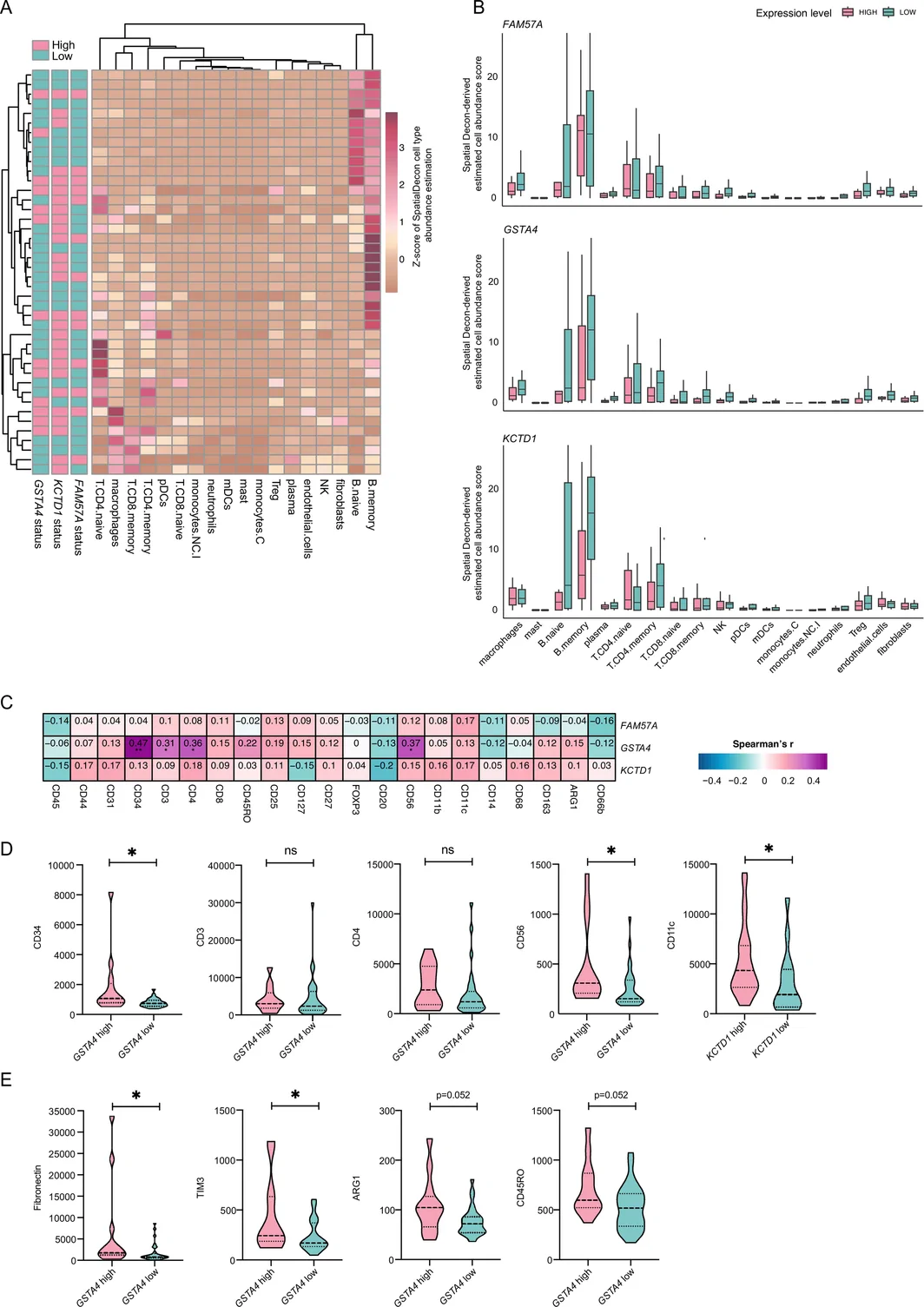
FAM57A, GSTA4 and KCTD1 expression in immune compartment is not strongly associated with altered immune infiltration patterns. (A) Immune cell abundance scores, estimated by Spatial Decon algorithm from RNA seq data, in the CD45^+^ immune compartment of 42 metastatic melanoma lymph node biopsies from 31 patients, coupled with marker expression status as determined in the survival analysis (B) Immune cell abundances estimated by Spatial Decon algorithm compared between high and low expression groups using either unpaired *t*‐test or Mann–Whitney *U*‐test, depending on the dataset distribution, evaluated by Shapiro–Wilk test. (C) Heatmap showing Spearman's correlation between the gene expression of PSP biomarkers and immune cell marker protein expression in the immune compartment. (D) Immune cell marker protein levels between PSP biomarker high and low groups in the immune compartment. Protein levels are reported as normalized counts. (E) Expression of protein markers related to different immune cell states between GSTA4 high and low groups. Protein levels are reported as normalized counts. Comparisons in this Figure were performed at the sample/compartment level, with samples from the same patient treated as separate observations. These analyses are exploratory and should not be interpreted as patient‐level comparisons.

All biomarkers displayed weak trends of lower immune infiltration, especially of B cells, evidenced by negative correlation with CD45 and CD20 protein levels, analysed by NanoString GeoMX (Figure 4C). The *GSTA4* expression correlated significantly with CD34, CD3, CD4 and CD56 protein levels (Figure 4C), of which CD34 and CD56 showed statistically significant difference between *GSTA4* high and low groups (Figure 4D) that were defined in the survival analysis (Figure 3D,E). CD34 was negatively linked to survival while CD56 was not linked to survival (Figure S4D). Interestingly, the weak trend of positive correlation between *KCTD1* and CD11c (Figure 4C) was further supported by increased CD11c expression in *KCTD1* high group (Figure 4D). However, CD11c was not significantly linked to survival (Figure S4D). Upon inspection of immune state markers, *GSTA4* levels were associated with fibronectin, TIM3, ARG1 and CD45RO levels, suggesting an immune activated but exhausted and suppressive environment in the immune cell compartment of metastatic melanoma lymph node biopsies (Figure 4E). Only ARG1 was negatively linked to survival (Figure S4E). These findings suggest that the clinical associations of the biomarkers in the immune compartment are unlikely to be primarily driven by differences in overall immune infiltration, and may instead reflect differences in immune cell functional states.

### 3.5 *FAM57A* , *GSTA4* And *KCTD1* Are Associated With Altered Transcriptional Programs Related to Antigen Presentation, Immune Effector Functions and Epigenetic Regulation

To characterize molecular patterns associated with biomarker‐high and biomarker‐low immune compartments, differential gene expression analysis was performed. Compared to *FAM57A*‐low compartments, *FAM57A*‐high compartments showed increased expression of genes with metabolic and transport functions (*SLC35F5*, *ALOX12B*, *EXOC3L1*) and reduced expression of immune‐related genes (*STAT1*, *GZMB*, *CCL2*, *LYZ*, *IFI44L*) (Figure 5A, upper panel). Reactome pathway analysis of up‐ and downregulated genes revealed that only downregulated genes showed significantly enriched pathways, mainly related to protein translation and ribosome biogenesis, suggesting reduced translational activity (Figure 5B, upper panel). Furthermore, neutrophil degranulation and cross‐presentation of soluble exogenous antigens (Table S2) pathways were enriched among downregulated genes. Gene Set Enrichment Analysis (GSEA) showed a negative enrichment score of IL2‐STAT5, IL6‐JAK‐STAT3, IFNy, TNFa via NF‐κB, complement, allograft rejection and inflammatory response, indicating higher activity of these immune‐related pathways in *FAM57A*‐low compartments (Figure 5C, upper panel, Figure S5A). Furthermore, MYC targets v1 and v2, MTORC1 signalling, PI3K–AKT–mTOR pathway, apoptosis, oxidative phosphorylation, ROS pathway, and unfolded protein response gene sets showed negative enrichment, indicating higher pathway activity in *FAM57A*‐low samples.

**FIGURE 5:**
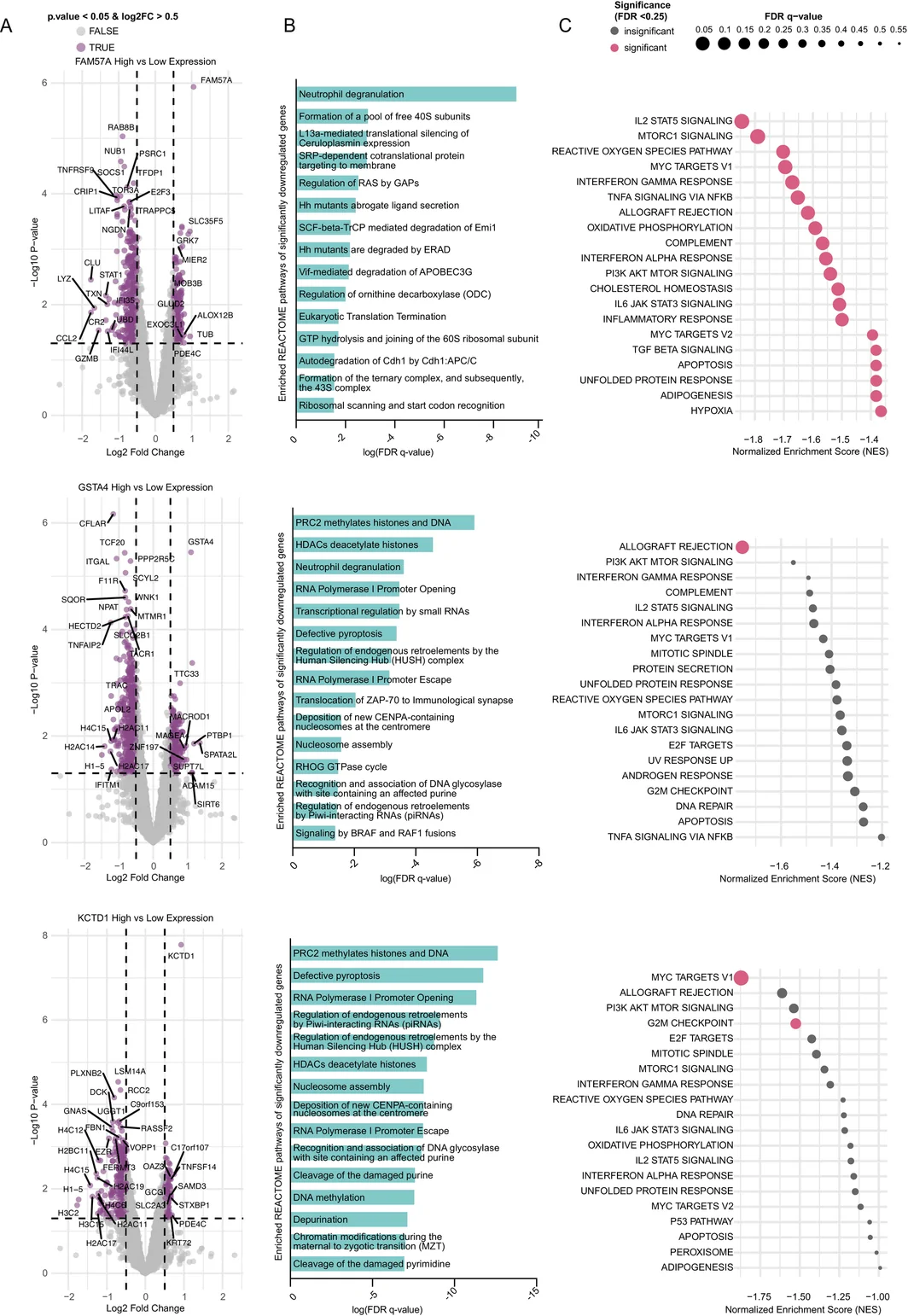
*FAM57A*, *GSTA4* and *KCTD1* are associated with altered transcriptional programs related to antigen presentation, immune effector functions and epigenetic regulation (A) Volcano plots showing Log_2_ Fold Change between biomarker high and low expression groups in the immune compartment of 42 metastatic melanoma lymph node biopsies from 31 patients. Top differentially expressed genes with Log(FC) > 0.5 or < −0.5 and *p* < 0.05 are highlighted with purple. (B) Significantly enriched Reactome pathways of the significantly downregulated genes in biomarker high vs. low expression groups (C) Gene Set Enrichment Analysis (GSEA) of biomarker high vs. low expression groups. Negative normalized enrichment score indicates enrichment of the gene set in the biomarker low expression group. Pink dots indicate significantly enriched (FDR < 0.25) gene set and the size of the dot represents the FDR q‐value with larger dot being more significant.

Differential expression analysis between *GSTA4*‐high and *GSTA4*‐low immune compartments revealed increased expression of genes associated with epigenetic modulation (*MACROD1*, *SIRT6*, *MAGEA4*) and reduced expression T cell and apoptosis related genes (*CFLAR*, *TRAC*, *ITGAL*, *TNFAIP2*) as well as histone genes, in *GSTA4*‐high compartments (Figure 5A, middle panel). The downregulated genes were enriched in reactome pathways related to chromatin remodelling, transcriptional regulation and ZAP70 translocation indicating altered transcriptional patterns and T cell activation (Figure 5B, middle panel). The allograft rejection gene set, comprising genes involved in innate and adaptive immune responses, showed negative enrichment in *GSTA4*‐high compartments (Figure 5C, middle panel, Figure S5A).

Interestingly, differential expression analysis between *KCTD1*‐high and *KCTD1*‐low immune compartments revealed increased expression of genes associated with T cell activation and signal transduction (*TNFSF14* [32], *SLC2A3* [33], *PDE4C* [34], SAMD3 [35]) and reduced expression of genes related to antigen processing, adhesion and migration related genes (*UGGT1*, *FERMT3*, *PLXNB2*) as well as histone genes in *KCTD1*‐high compartments (Figure 5A, lower panel). Reactome pathways related to epigenetic repression were significantly enriched among the downregulated genes. MYC v1 targets and G2M checkpoint gene sets were enriched in *KCTD1*‐low compared to *KCTD1*‐high, indicating higher proliferative signalling in *KCTD1*‐low samples (Figure 5C, lower panel, Figure S5A).

These results suggest that high expression of *FAM57A*, *GSTA4* and *KCTD1* in the immune compartments of lymph nodes is associated with altered transcriptional programs related to epigenetic regulation, immune effector functions and antigen presentation.

### 3.6 Co‐Expression of *FAM57A* , *GSTA4* , and *KCTD1* Associates With PI3K/AKT/mTORC Signalling and Poor Survival Outcomes in Metastatic Melanoma Immune Compartments

Although PI3K/AKT/mTORC‐related pathways were enriched at the transcriptomic level only in relation to *FAM57A*, we hypothesized that the protein‐level signalling activity may still be related to the expression of all biomarkers. To test this, phosphorylation events linked to these signalling cascades were examined in the CD45^+^ immune compartment of metastatic melanoma lymph node samples.

*FAM57A* expression was significantly associated with increased phosphorylation of PRAS40 (T246) and p90RSK (T359/S363), suggesting enhanced AKT–mTORC1 and MAPK pathway activity (Figure 6A). Phosphorylation of GSK3α/β (S21/S9) correlated positively with *FAM57A* levels but not statistically significantly with the high/low status (Figure 6A). Phospho‐AKT1 (S473) correlated positively with *KCTD1* but did not demonstrate significant phosphorylation differences between high and low expression groups (Figure 6B). *GSTA4* expression correlated significantly with elevated levels of PLCG1, and phosphorylation of Tuberin (T1462) and AKT1 (S473), suggesting activation of AKT–mTORC1 (Figure 6C). Together, these findings illustrate distinct signalling phenotypes for each biomarker: *FAM57A* and *GSTA4* are linked to increased AKT–mTORC1 and MAPK activity, whereas transcriptomic *KCTD1* shows less pronounced signalling changes consistent with its transcriptomic profile.

**FIGURE 6:**
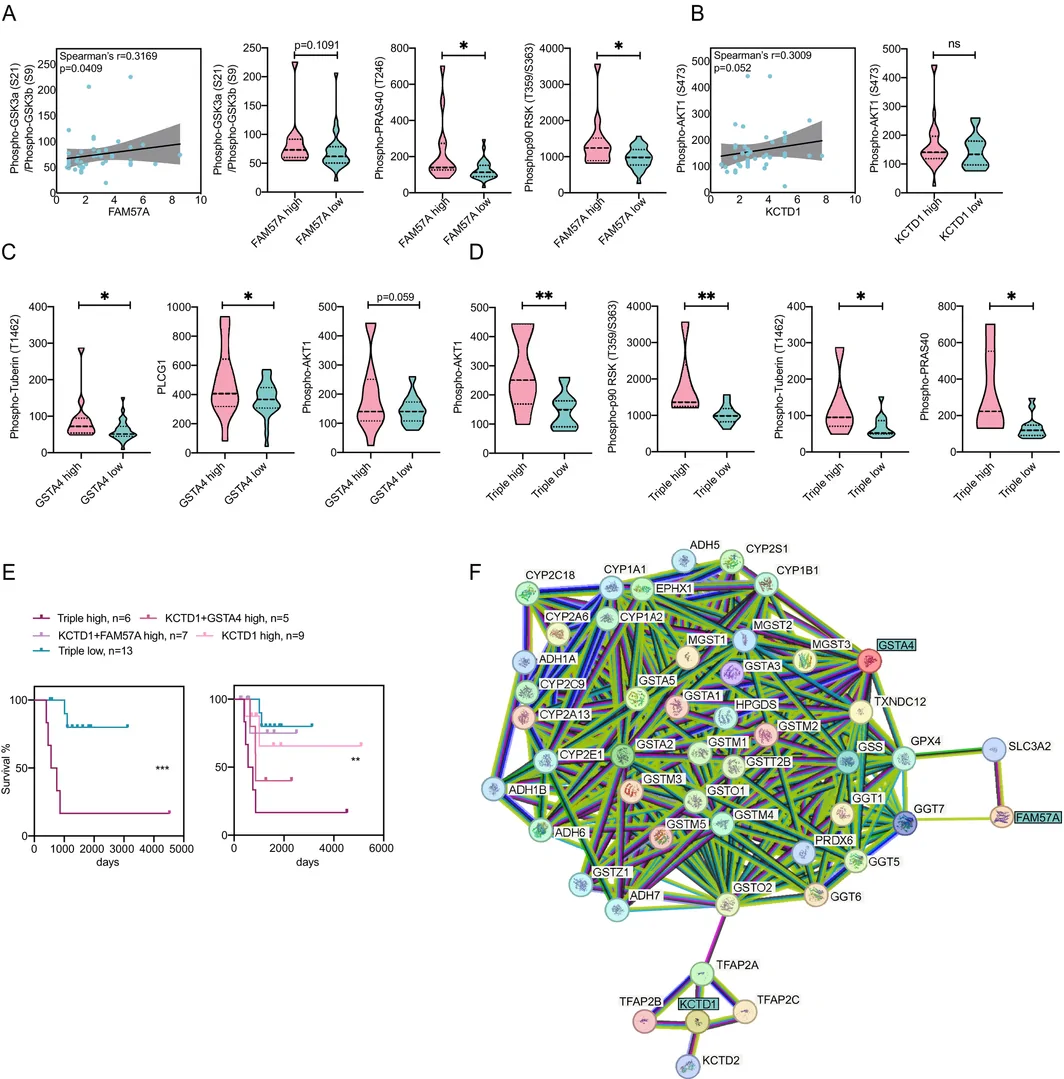
Co‐Expression of *FAM57A*, *GSTA4* and *KCTD1* marks immune compartments with increased PI3K/AKT/mTORC signalling and associates with reduced survival outcomes in metastatic melanoma immune compartments. (A) Correlation plot of *FAM57A* with phospho‐GSK3a (S21)/3b (S9) and phosphorylation levels of multiple signalling components in immune compartment of metastatic melanoma lymph node biopsies (*n* = 42) as measured by NanoString GeoMX. (B) Correlation plot of *KCTD1* with phospho‐AKT1 (S473) and phosphorylation levels in immune compartment of metastatic melanoma lymph node biopsies (*n* = 42) as measured by NanoString GeoMX. (C) Phosphorylation levels of multiple signalling components in relation to *GSTA4* levels in immune compartment of metastatic melanoma lymph node biopsies (*n* = 42) as measured by NanoString GeoMX. (D) Phosphorylation levels of multiple signalling components in relation to triple high (*n* = 6) or triple low (*n* = 13) *FAM57A*, *GSTA4* and *KCTD1* levels in immune compartment of metastatic melanoma lymph node biopsies as measured by NanoString GeoMX. (E) Kaplan–Meier survival plots for different combinations of biomarker high and low statuses in immune compartment of metastatic melanoma lymph node biopsies (F) Full STRING network of physical and functional protein associations of *FAM57A*, *GSTA4* and *KCTD1*.

Given the shared signalling pathways associated with *FAM57A*, *GSTA4* and *KCTD1*, we stratified samples based on simultaneous high or low expression of all three biomarkers to assess whether their combined expression was associated with immune signalling and clinical outcomes. Triple high samples displayed statistically significantly elevated phosphorylation of key signalling proteins Phospho‐AKT1 (S473), Phospho‐p90 RSK (T359/S363), Phospho‐Tuberin (T1462), and Phospho‐PRAS40 (T246) compared to triple low samples (Figure 6D). This suggests that simultaneous high expression of *FAM57A*, *GSTA4* and *KCTD1* is associated with increased AKT‐mTORC1 and MAPK pathway activation in the lymph node immune compartment. Importantly, the triple high group exhibited markedly worse overall survival compared to the triple low group (Figure 6E), indicating that the simultaneous high expression of all three biomarkers identifies immune compartments associated with worse clinical outcomes.

To further explore potential interactions among these biomarkers, we performed protein–protein interaction analysis using the STRING database. The biomarkers formed an interconnected network via intermediate proteins, including components involved in glutathione metabolism, the transcription factor AP‐2 (TFAP2), and the amino acid transporter SLC3A2 (Figure 6F), suggesting that these biomarkers can be mapped to known functional interaction networks. Together, these data show that co‐expression of *FAM57A*, *GSTA4* and *KCTD1* identifies an immune compartment associated with increased AKT‐mTORC1 and MAPK signalling and poor prognosis.

## 4 Discussion

Although anti‐PD1 treatment has led to remarkable clinical benefit for metastatic melanoma patients, diverse resistance‐associated immune states continue to limit durable responses across patient populations. Although the DC‐CD8^+^ T‐cell axis has been extensively implicated in antitumor immunity, biomarkers associated with this axis remain limited. In our recent work, 129 poor survival predictor (PSP) biomarkers were identified as inversely associated with this axis, despite many lacking established roles in antitumor immunity. Here, we identified 28 PSP biomarkers that were significantly elevated in pre‐treatment samples from nonresponders in an integrated anti‐PD1 bulk RNA‐sequencing dataset. Several of these biomarkers have previously been implicated in cancer progression, metabolism, or intracellular signalling, with recent studies also suggesting roles for some in anti‐PD1 resistance and antitumor immunity. Notably, GPSM1 and TUB have been linked to dysfunctional immune response while GSTA4, RCOR2, and USP22 have been linked to melanoma immune evasion [36, 37, 38, 39, 40]. Rather than identifying these PSP biomarkers as part of novel resistance mechanisms, our findings link previously underappreciated PSP biomarkers to immune compartments characterized by established resistance‐associated features in metastatic melanoma, such as reduced antigen presentation‐related programs, decreased cytotoxic immune activity, immunosuppressive immune states, and activation of PI3K‐AKT‐mTORC/MAPK signalling [41, 42].

PSP biomarker expression was most strongly associated with the abundance of macrophage populations, particularly patterns reflecting M1/M2 macrophage balance, suggesting an association with altered myeloid states rather than broad changes in lymphocyte infiltration. Spatial compartment analysis further demonstrated that several PSP biomarkers showed differential expression across tumour and immune compartments, with some exhibiting modestly higher expression in tumour cells. Importantly, survival associations were compartment‐dependent, as certain biomarkers were prognostically relevant primarily within the tumour cell compartment, whereas others were associated with outcomes specifically in the immune cell compartment. Notably, *FAM57A*, *GSTA4* and *KCTD1* retained poor survival predictor characteristics when highly expressed in the CD45^+^ immune compartment of lymph node metastases. In contrast, these biomarkers were not statistically significantly associated with outcomes in the CD45^+^ immune compartment of melanoma skin metastases. Whether this reflects limited statistical power due to the smaller number of skin metastasis samples or biological differences in the immune cell composition and associated functional programs between lymph node and skin metastatic microenvironments remains unclear. Although the 28 PSP biomarkers correlated positively with immunosuppressive and negatively with immunostimulatory cell types in the integrated anti‐PD1 dataset, these associations were not replicated in the CD45^+^ immune compartment. As PSP biomarkers were expressed in both tumour and immune compartments, the bulk‐level infiltration associations may partly reflect biomarker expression in tumour cells. However, statistically significant survival associations in the tumour compartment were observed only for *ANKS6*, *FAM189B*, *HOXA11* and *KCTD15*, indicating that tumour cell expression may contribute to the bulk‐level infiltration patterns but does not fully explain the compartment‐specific prognostic associations of PSP biomarkers.

Further characterization of marker‐associated transcriptional programs revealed distinct pathway enrichment patterns. Although *FAM57A* lacks established direct functional annotation in Reactome, *FAM57A*‐stratified gene expression showed enrichment of pathways related to translation, immune activation, and signalling, consistent with broad PSP‐associated biological categories. *GSTA4* and *KCTD1* showed similar enrichment of immune, metabolic, and signalling‐related pathways. Notably, high *GSTA4* expression was associated with higher PLCG1 levels, while *GSTA4*‐low samples showed enrichment of ZAP70 translocation‐related gene sets, suggesting differential association with components of TCR signalling pathways. However, the cellular context of these signals cannot be determined from the present dataset. High biomarker expression levels were linked to increased activating phosphorylation of mTORC1 pathway components, with a more pronounced effect observed in samples with high expression of all biomarkers. Previous reports have linked FAM57A (also known as CT120) to Raf/Mek/ERK and PI3K/AKT signalling pathways in NIH3T3 cells [43]. Although mechanistic role of FAM57A remains incompletely understood, it has been associated with amino acid transport, a process associated with mTORC1 regulation involving FAM57A interaction partner SLC3A2 [44]. Both GSTA4 and its interaction partner GPX4 have been associated with the mTORC1 pathway in various tumour cells [45, 46]. KCTD1 has not been directly linked to signalling pathways in tumour or immune cells but it is known to be a repressor of TFAP2 [47] and has been shown to interact with c‐MYC to promote transcriptional activation of PD‐L1 in hepatocellular carcinoma [48].

This study has a few important limitations. Although the initial biomarker discovery was performed in large integrated cohorts, the relatively modest size of the spatial validation cohort following sample stratification limited statistical power. In addition, the use of an optimal log‐rank cut‐point within a predefined interquartile range may introduce optimistic bias, particularly given the narrow expression range of *FAM57A*, *GSTA4* and *KCTD1*. Although spatial compartment analyses identified compartment‐specific associations, the compartment‐level resolution does not provide sufficient resolution to definitively identify cell types expressing these biomarkers. Although PSP biomarkers are associated with the DC‐CD8^+^ T cell axis through their correlation with reference biomarkers *CD74* and *CD8A*, the rationale should not be interpreted as exclusively identifying molecules involved in direct DC‐CD8^+^ T cell interactions. Importantly, this rationale does not exclude the possibility that PSP markers (including FAM57A, GSTA4 and KCTD1) may be expressed by multiple cell types or that their predominant cellular source lies outside the DC‐CD8^+^ T cell axis. Instead, regardless of the source of the expression, these biomarkers may impact the axis or mark immune states linked with the functionality of the axis, as indicated, for example, by the negative correlation with *CD74*, *CD8A* and cytotoxic signature in the TME. Consequently, the precise cellular sources and functional contexts of these biomarkers remain difficult to resolve with the current approaches. The compartment‐specific associations observed for *FAM57A*, *GSTA4* and *KCTD1* further emphasize the importance of spatial context in defining their clinical relevance. Moreover, the correlative nature of this study precludes conclusions regarding the mechanistic roles of these biomarkers. It therefore remains unclear whether these biomarkers contribute directly to immunosuppressive states of immune cells within the TME or instead reflect compensatory responses arising in already dysfunctional immune environments. Future studies employing larger independent cohorts for single‐cell approaches or highly multiplexed spatial imaging, together with functional validation, will be valuable for resolving the cellular origin of these biomarkers and elucidating their mechanistic roles within the TME, particularly in relation to the DC‐CD8^+^ T cell axis. Combining spatial and functional studies will be important to determine how PSP‐associated programs may contribute to anti‐PD1 resistance and whether they can support patient stratification.

## Author Contributions

S.A.K. conceptualized and designed the study, led and performed formal analysis and interpretation of transcriptomic and NanoString GeoMX spatial data. C.R.F. designed and implemented the Spatial GeoMX Nanostring project. S.A.K. also contributed to the acquisition of NanoString GeoMx data with technical assistance. R.E.K. organized biobank participants. E.‐M.B. provided sample pathological assessments. S.A.K., M.S., and C.R.F. wrote the first draft of the manuscript. C.R.F. and M.S. contributed to planning the melanoma biobank study and collected clinical data. I.M. collected clinical data. C.R.F. contributed to research funding acquisition for the project. M.S. and C.R.F. supervised the study. All authors contributed to the final review.

## Funding

S.K. received support from Suomen Kulttuurirahasto (Finnish Cultural Foundation; grant nos. 00240691 and 00260112) and the Turku Doctoral Programme of Molecular Medicine. This project, including the work of C.R.F., was mainly supported by the Academy of Finland (grant nos. 330876, 336530, and 358209), Jane ja Aatos Erkon Säätiö (grant nos. 5957‐12539 and 7180‐2BD08), Sigrid Juséliuksen Säätiö (grant nos. 220022 and 230026), Turun Yliopistosäätiö (Turku University Foundation; grant no. 081497), and the InFLAMES Flagship (grant nos. 337530, 357910, and 358823).

## Ethics Statement

The spatial analyses of human metastatic melanoma tissues were conducted in accordance with the Declaration of Helsinki. The use of archived biobank samples was approved for research purposes, with patient consent obtained according to biobank procedures. Ethical approval was provided by the Scientific Steering Ethical Committee of Auria Biobank under decision AB22‐4803, dated 27 April 2022. Sample use complied with the Finnish Biobank Act (688/2012).

## Conflicts of Interest

The authors declare no conflicts of interest.

## Supporting information


**Figure S1:** Analysis of gene expression between responders and non‐responders reveals response‐associated poor survival predictors (PSP). (A) Box plots showing gene expression levels for each PSP gene between responders and non‐responders. Statistical significance of difference was analysed using Mann–Whitney *U*‐test for each gene. Box indicates middle 50% while central line indicates median and whiskers represent the range.
**Figure S2:** CIBERSORTx immune abundance estimations for many immune cell types are not linked to anti‐PD1 response in integrated anti‐PD1 dataset. Violin plots showing immune cell abundance levels, estimated by CIBERSORTx, in responders and non‐responders of the integrated anti‐PD1 dataset.
**Figure S3:** *ARHGAP42* shows differential survival trend in tumour and immune compartments. (A) Kaplan–Meier survival plots of *ARHGAP42* in tumour (S100B/PMEL17^+^) compartment of 31 metastatic melanoma lymph node biopsies from 31 patients analysed using NanoString GeoMX and log‐rank test. (B) Kaplan–Meier survival plots of *FAM57A* and *KCTD1* in immune (CD45^+^) compartment of 31 metastatic melanoma lymph node biopsies from 31 patients analysed using NanoString GeoMX and log‐rank test.
**Figure S4:** *FAM57A*, *GSTA4* and *KCTD1* expression in immune compartment is not strongly associated with altered immune infiltration patterns. Immune cell marker protein levels compared between (A) *FAM57A* (B) *GSTA4* (C) *KCTD1* high and low groups in the immune compartment. Using either unpaired *t*‐test or Mann–Whitney *U*‐test, depending on the dataset distribution, evaluated by Shapiro–Wilk test. (D) Kaplan–Meier survival plots of immune cell phenotypic markers in immune (CD45^+^) compartment of 42 metastatic melanoma lymph node biopsies from 31 patients analysed using NanoString GeoMX and log‐rank test. (E) Kaplan–Meier survival plots of immune cell functional markers in immune (CD45^+^) compartment of 42 metastatic melanoma lymph node biopsies from 31 patients analysed using NanoString GeoMX and log‐rank test.
**Figure S5:** *FAM57A*, *GSTA4* and *KCTD1* expression are associated with distinct hallmark gene sets. Representative plots of the most enriched hallmark gene sets from Gene Set Enrichment Analysis.
**Table S1:** Figure 4 SpatialDecon Abundances.
**Table S2:** Figure 5 Reactome pathways.

**Data S1:** sji70153‐sup‐0002‐DataS1.xlsx.

## Data Availability

RNA‐seq data from the TCGA‐SKCM cohort can be accessed through the Genomic Data Commons portal (https://portal.gdc.cancer.gov), with normalized data also available through UCSC Xena (https://xenabrowser.net/). Publicly available RNA‐seq datasets from immune checkpoint inhibitor‐treated metastatic melanoma cohorts were retrieved from the repositories reported in the original studies, including GEO, SRA, ENA and dbGaP. The accession numbers are as follows: Liu et al. cohort [24], phs000452.v3.p1; Hugo et al. cohort [8], GSE78220; Riaz et al. cohort [26], GSE91061 and SRP094781; Gide et al. cohort [25], PRJEB23709. Spatial whole transcriptome data from melanoma patients in the Auria cohort are available upon reasonable request, and according to data use guidelines.
