# Supplementary material for: Spatial Transcriptomic and Proteomic Analysis Identifies Novel Immune‐Associated Biomarkers of Anti‐PD1 Response in Metastatic Melanoma
Source: Scand J Immunol. 2026 Oct 21;104(4):e70153. doi: 10.1111/sji.70153 (PMC13687651; doi:10.1111/sji.70153)
Supplement: Supplementary file 1 [file SJI-104-e70153-s002.docx]

**Supplementary Figure Legends**

**Supplementary Figure 1:** Analysis of gene expression between responders and non-responders reveals response-associated poor survival predictors (PSP). A) Box plots showing gene expression levels for each PSP gene between responders and non-responders. Statisical significance of difference was analysed using Mann-Whitney U-test for each gene. Box indicates middle 50% while central line indicates median and whiskers represent the range.

**Supplementary Figure 2: CIBERSORTx immune abundance estimations for many immune cell types are not linked to anti-PD1 response in integrated anti-PD1 dataset.** Violin plots showing immune cell abundance levels, estimated by CIBERSORTx, in responders and non-responders of the integrated anti-PD1 dataset.

**Supplementary Figure 3:**  ***ARHGAP42* shows differential survival trend in tumor and immune compartments. A)** Kaplan-Meier survival plots of *ARHGAP42* in tumor (S100B/PMEL17^+^) compartment of 31 metastatic melanoma lymph node biopsies from 31 patients analysed using NanoString GeoMX and log-rank test. **B)** Kaplan-Meier survival plots of *FAM57A* and *KCTD1* in immune (CD45^+^) compartment of 31 metastatic melanoma lymph node biopsies from 31 patients analysed using NanoString GeoMX and log-rank test.

**Supplementary Figure 4**: ***FAM57A*, *GSTA4* and *KCTD1* expression in immune compartment is not strongly associated with altered immune infiltration patterns.** Immune cell marker protein levels compared between **A) *FAM57A* B) *GSTA4* C) *KCTD1*** high and low groups in the immune compartment. using either unpaired t-test or Mann-Whitney U-test, depending on the dataset distribution, evaluated by Shapiro-Wilk test. **D)** Kaplan-Meier survival plots of immune cell phenotypic markers in immune (CD45^+^) compartment of 42 metastatic melanoma lymph node biopsies from 31 patients analysed using NanoString GeoMX and log-rank test. **E)** Kaplan-Meier survival plots of immune cell functional markers in immune (CD45^+^) compartment of 42 metastatic melanoma lymph node biopsies from 31 patients analysed using NanoString GeoMX and log-rank test.

**Supplementary Figure 5**: ***FAM57A*, *GSTA4* and *KCTD1* expression are associated with distinct hallmark gene sets.** Representative plots of the most enriched hallmark gene sets from Gene Set Enrichment Analysis

**Supplementary Figures**

**Supplementary Figure 1**

**Supplementary Figure 2**

**Supplementary Figure 3**

**Supplementary Figure 4**

**Supplementary Figure 5**

**Supplementary Table Legends**

**Supplementary Table 1 - Figure 4 SpatialDecon Abundances**

**Supplementary Table 2 - Figure 5 Reactome pathways**
